# Is creatine kinase a valid aid in early cardiac muscle damage detection during treatment for childhood acute lymphoblastic leukemia? A case report

**DOI:** 10.3389/fonc.2026.1668068

**Published:** 2026-05-01

**Authors:** Grazia Fazio, Jari Intra, Orsola Montini, Laura Rachele Bettini, Giacomo Gotti, Alessandra Sala, Silvia Bungaro, Giovanni Cazzaniga, Carmelo Rizzari, Adriana Balduzzi, Marco Casati

**Affiliations:** 1 Tettamanti Center, Fondazione IRCCS San Gerardo dei Tintori, Monza, Italy; 2 School of Medicine and Surgery, University of Milano-Bicocca, Milan, Italy; 3 Clinical Chemistry Laboratory, Fondazione IRCCS San Gerardo dei Tintori, Monza, Italy; 4 Pediatrics, Fondazione IRCCS San Gerardo dei Tintori, Monza, Italy; 5 Laboratorio Ultraspecialistico Patologia Clinica-Ematologia, Fondazione IRCCS San Gerardo dei Tintori, Monza, Italy; 6 Medical Genetics, Fondazione IRCCS San Gerardo Dei Tintori, Monza, Italy

**Keywords:** ALL, biomarkers, cardiotoxicity, chemotherapy, childhood cancer, creatine kinase, leukemia

## Abstract

Creatine kinase (CK) is an enzyme that plays a pivotal role in various physiological processes, including metabolism and muscle function. This enzyme is found in high concentrations in skeletal muscle and the myocardium, with lower levels present in the brain. Increases in CK values have been observed in muscle damage and neuromuscular disorders. The potential for cardiotoxicity resulting from the administration of chemotherapeutic agents has been identified in subjects affected by hematological or solid tumors. In the present work, we describe the case story of a child diagnosed with acute lymphoblastic leukemia who exhibited two episodes of asymptomatic, markedly elevated CK values, identified during chemotherapy treatment. Our aim was to investigate the potential of CK as a biomarker for the early detection of cardiac muscle damage during therapy.

## 1 Introduction

Acute lymphoblastic leukemia (ALL) is the most common pediatric malignancy, representing approximately 25% of cancer diagnoses. It has been established that ALL accounts for 75-80% of pediatric acute leukemias, with an incidence of 3–4 cases per 100,000 children younger than 15 years. Children aged between 2 and 5 years old are most commonly affected, with a higher prevalence in males (1, 2). Late adolescence, comprising patients aged 15 to 19, is less affected than childhood or early adolescence (9–14 years old); however, these adolescents are more likely to exhibit high-risk features and inferior outcomes (1). ALL is characterized by uncontrolled T- or B-cell proliferation, whereby abnormal and immature cells replace healthy cells and their progenitors in the bone marrow, spleen, liver, and lymph nodes. In recent decades, the overall 5-year survival probability achieved in children diagnosed with ALL has exceeded 90% (2, 3). Several factors have been identified as crucial to increasing the survival rate, including the sub-classification of ALL cases through genomic methodologies, new molecular findings, risk stratification, and chemotherapy-intensified regimens, along with improvements in supportive care (2, 3). This has given rise to an emerging interest in preserving health and quality of life among childhood ALL survivors, with late cardiotoxicity being one of the most severe non-hematological treatment-related complications. A comparative analysis of the morbidity and mortality rates in childhood ALL survivors and their peers reveals that the former demonstrate a higher incidence of cardiovascular complications. It has been demonstrated that cardiotoxic effects can occur during treatment, with a concomitant impact on patient outcomes (4–7). Several biomarkers have been used for the identification of cardiac dysfunction and the prediction of cardiotoxic risk. Among these, troponin and N-terminal pro-brain natriuretic peptide (NT-pro-BNP) levels have been reported to be associated with left ventricular (LV) remodeling (4, 6).

Creatine kinase (CK) is an enzyme that plays a pivotal role in various cellular physiological processes, including cellular metabolism and muscle function. This enzyme is found in high concentrations in skeletal muscle and the myocardium, with lower levels present in the brain. Increases in CK values have been observed in several conditions, including muscle damage from strenuous exercise, myocardial infarction, skeletal muscle injuries, and neuromuscular disorders such as muscular dystrophy, metabolic and inflammatory myopathies, and central or peripheral neuropathies. Elevated CK values have also been detected in non-neuromuscular conditions, including pulmonary infarction and edema, hypothyroidism, following high alcohol consumption, and in response to specific drug classes, including statins, antipsychotics, antivirals, and beta-blockers (8, 9). Chemotherapeutic agents, in particular anthracyclines, nucleotide synthesis inhibitors, alkylating agents, and tyrosine kinase inhibitors, have been implicated in cardiotoxicity events occurring in subjects affected by hematological or solid tumors (6, 9).

In the present work, we describe the case of a child diagnosed with ALL who exhibited two asymptomatic, markedly elevated CK values during routine chemical analyses, performed periodically during chemotherapy treatment. The objective of this report was to examine current knowledge on the potential utilization of CK as a biomarker to facilitate early detection of cardiac muscle damage during chemotherapy, thus going beyond a mere clinical report.

## 2 Case description

In 2024, a 16-year-old female presented with hemorrhagic diathesis in the upper and lower limbs associated with mild fever and diffuse bone and muscle pain. The complete blood count revealed a white blood cell (WBC) count of 5.18x10^9^/L (reference range: 5.0-13.0x10^9^/L) with 51.2% lymphocytes and 31.5% monocytes, hemoglobin (Hb) of 85 g/L (reference range: 120–160 g/L), and platelet count (PLT) of 67x109/L (reference range: 140–440x109/L). The peripheral blood (PB) smear revealed a blast population of 18%. Biochemical analysis was performed on Cobas 8000 analyzers (Roche Diagnostics, Mannheim, Germany). The results were as follows: glucose 85 mg/dL (reference range: 70–110 mg/dL), C-reactive protein (CRP) 8.8 mg/L (reference range: <5 mg/L), and elevated serum lactic dehydrogenase (LDH) 391 U/L (reference range: 135–214 U/L). Upon admission to the Pediatric Hematology-Oncology Unit of the Foundation IRCCS San Gerardo dei Tintori (Monza, Italy), a bone marrow (BM) aspiration was conducted, revealing a monomorphic invasion of atypical elements (95%) that exhibited the morphological characteristics of lymphoblasts. The patient was diagnosed with B-cell precursor ALL and was treated in accordance with the Associazione Italiana Ematologia Oncologia Pediatrica (AIEOP) and Berlin-Frankfurt-Münster (BFM) ALL 2017 protocol (EudraCT Number: 2016-001935-12). Baseline cardiac status was normal (pre-anthracycline ECG and echocardiogram). The timeline from diagnosis is schematically shown in **Table 1**. The genetic analysis indicated the presence of the ETV6::RUNX1 fusion gene, encoded by the translocation t(12;21), and a hyperdiploid profile, with the following karyotype: 73~77,XX,-?X,-?3,+5,-?11,+?19,+21,+22,+mar,inc[cp6]/46,XX[4]. Despite the presence of ETV6::RUNX1, typically associated with a favorable prognosis (10), the patient demonstrated resistance to the induction phase IA at day +33 (time point 1, TP1). This phase involves the administration of four doses of daunorubicin administered at 30 mg/m^2^/dose, resulting in the persistence of lymphoid blasts (7%). The molecular Minimal Residual Disease (MRD) at TP1 was positive for both markers in the range of 10^-1^ (marker 1 TRG Vg3 Jg1.3/2.3 = 1.6x10_–1_ with Quantitative Range (QR)=1x10_–4_ and IGK VK2 JK3 = 1.8x10_–1_ with QR = 5x10^-4^), as reported in **Table 1**.

**Table 1:** Timeline and the applied multidisciplinary clinical interventions.

Clinical phase	Laboratory & diagnostic findings	Therapeutic interventions
Initial Presentation	WBC: 5.18x10^9^/L; Hb: 85 g/L; PLT: 67x10^9^/L; LDH: 391 U/L; CRP: 8.8 mg/L PB Smear: 18% blasts.	Admission and baseline cardiac screening (Normal ECG/Echocardiogram)
Diagnosis	BM Aspirate: 95% lymphoblasts. Genetics: ETV6::RUNX1 fusion; t(12;21); Hyperdiploid karyotype (73~77,XX)	Initiation of AIEOP-BFM ALL 2017 protocol
Induction (Phase IA)	Day+33 (TP1): Residual blasts (7%); MRD 10^-^¹ (TRG Vg3 Jg1.3/2.3 and IGK VK2 JK3)	Daunorubicin (4 doses at 30 mg/m²/dose)
Cardiac alterations	CK: 1,053 U/L; hs-cTnT: 148 ng/L ECG: Sinus tachycardia, T-wave alterations Echo: EF 60%, normal contractility.	Supportive therapy
Induction (Phase IB)	Severe neutropenia (WBC: 0.42x10^9^/L). Stool Culture: Rotavirus (+) Blood cultures: Negative Molecular MRD negative	Cyclophosphamide, 6-Mercaptopurine, ARA-C. Empirical antibiotics (Piperacillin/Tazobactam, Amikacin); Molecular CR achieved
Consolidation (Block 1)	Recurrent CK elevation: 532 U/L	Modified Block 1
Consolidation (Block 2)	Pre/Post-infusion CK and Troponin: Within normal range	Substitution of Daunorubicin with Liposomal Doxorubicin
Pre-Transplant Phase	Persistent Molecular CR (MRD negative)	Blinatumomab (15 µg/m²/day for two 28-day cycles).
Post HSCT	No cardiac abnormalities (normal ECG/echocardiography) Molecular MRD negative (up to +12months post HSCT)	Regular follow-up: therapeutic management and monitoring. Continuous Molecular CR

ARA-C, Cytarabine; BM, Bone Marrow; CK, Creatine Kinase; CR, Complete Response; CRP, C-Reactive Protein; ECG, Electrocardiogram; EF, Ejection Fraction; Hb, Hemoglobin; hs-cTnT, High-sensitivity Cardiac Troponin T; HSCT, Hematopoietic Stem Cell Transplant; LDH, Lactic Dehydrogenase; MRD, Minimal Residual Disease; PB, Peripheral Blood; PLT, Platelets; TP1, Time Point 1; WBC, White Blood Cells.

**Figure 1:**
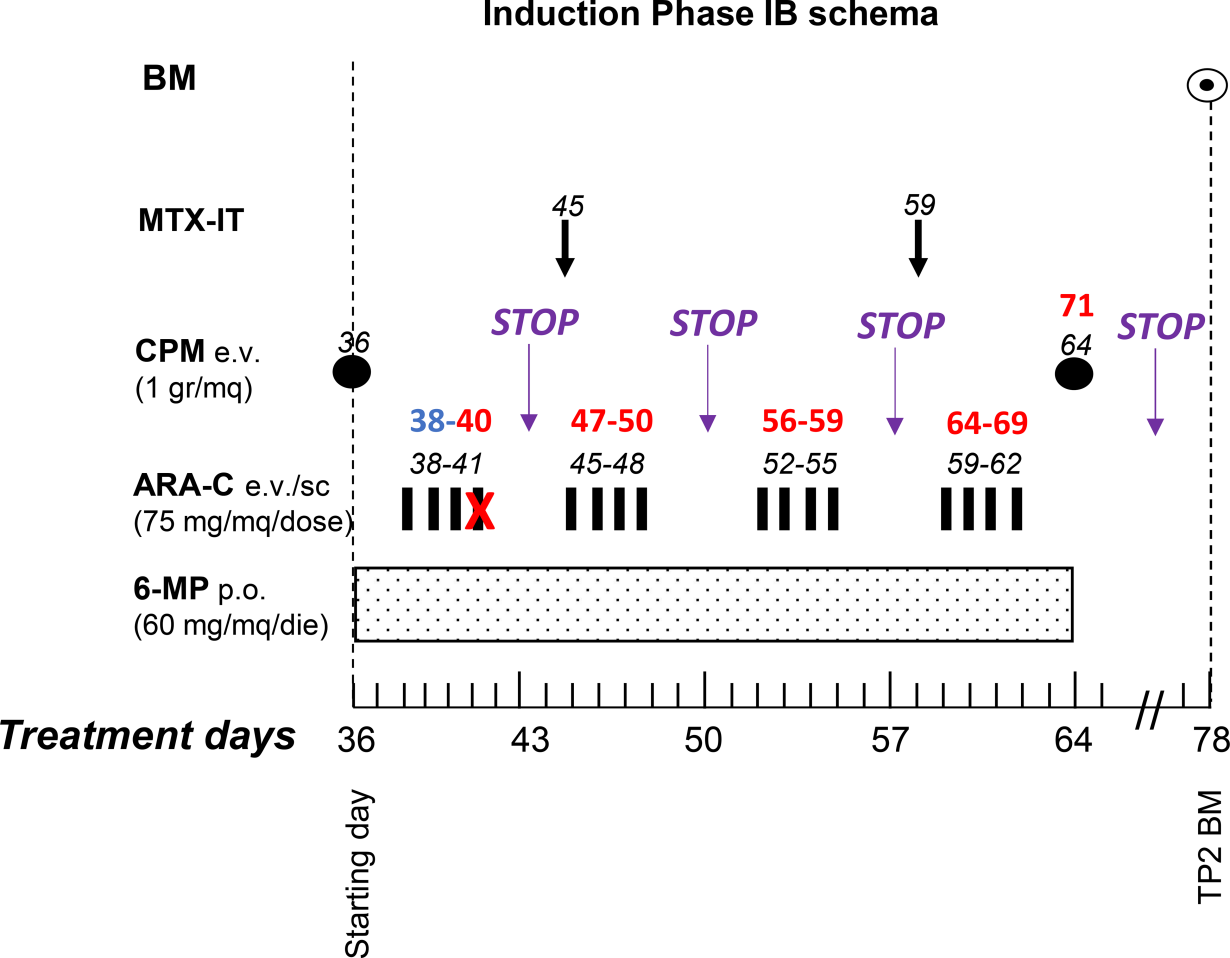
Induction phase IB schema. The plot shows the chemotherapeutic drugs administered to our patient during the treatment block, according to the AIEOP-BFM 2017 protocol, including interruptions due to toxicity and infections as described in the text. Abbreviations: Bone marrow aspirate (BM), intrathecal methotrexate (MTX-IT), cyclophosphamide (CPM), cytarabine (Ara-C), and 6-mercaptopurine (6-MP), time point 2 (TP2).

The chemotherapy treatment proceeded with phase IB, consisting of cyclophosphamide, 6-mercaptopurine, and cytosine arabinoside (ARA-C) (**Figure 1**; **Table 1**). During the subsequent neutropenic phase (Hb 100 g/L, PLT 77 x 10^9^/L, and WBC 0.42 x 10^9^/L), the patient was hospitalized due to a deterioration in clinical conditions. The patient was administered supportive therapy, and the fourth dose of ARA-C was withheld due to the onset of fever and diarrhea. Central venous catheter and peripheral blood cultures were negative, while stool cultures tested positive for Rotavirus. Empirical antibiotic therapy (piperacillin-tazobactam and amikacin) was initiated. Following an improvement in the patient’s clinical condition, chemotherapy was reinitiated and continued for a period of three weeks. During this period, following two episodes of fever, antibiotic therapies with vancomycin and ceftazidime were administered; however, blood cultures remained consistently negative.

**Figure 2:**
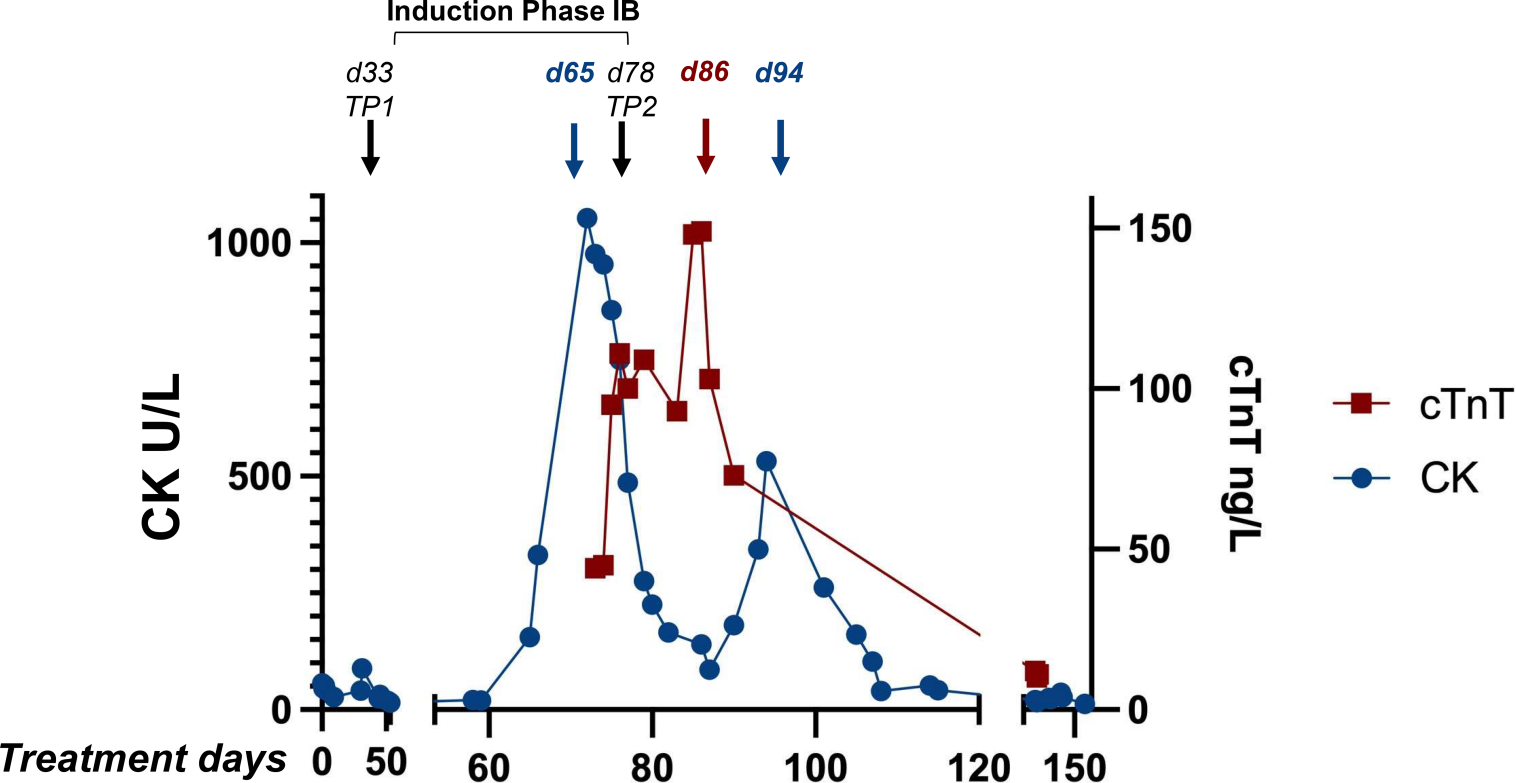
Creatine kinase and troponin elevations. The diagram shows the increases in CK and troponin detected at the end of the induction phase IB. Abbreviations: Creatine kinase (CK), cardiac troponin T (cTnT), time point 1 (TP1), time point 2 (TP2), and peak (pk).

At the conclusion of the third ARA-C block, CK serum levels exhibited a progressive increase, attaining a maximum value of 1,053 U/L, from an initial value of 155 U/L (reference value: <170 U/L) (**Figure 2**; **Table 1**). Following the completion of the second cyclophosphamide infusion, the patient presented with symptoms of general malaise and frontal headache. The electrocardiogram (ECG) revealed sinus tachycardia with non-specific T-wave alterations on lateral derivations. The measurement of high-sensitivity cardiac Troponin T was conducted subsequent to the detection of the elevated CK values, showing an escalating trend from 44 to 148 ng/L (reference value: < 14 ng/L). Considering the laboratory findings and the sinus tachycardia, a second ECG was performed. Echocardiography excluded kinesis and contraction deficiencies, showing an ejection fraction (EF) of 60%. A complete molecular response (CR) was observed (both MRD markers were negative with QR = 1x10^-4^) at the end of phase IB. In view of the complications, a modified consolidation with block 1 was initiated. During the standard laboratory follow-up, the CK value increased once more to 532 U/L, with a subsequent gradual normalization within a few days (**Figure 2**, **Table 1**). Given the previous findings, although no specific causative drug was identified as responsible for the elevation of CK and troponin levels, daunorubicin was substituted with liposomal doxorubicin to ensure greater cardioprotection during block 2. This decision was guided by a multidisciplinary cardio-oncology discussion in accordance with the protocol. Cardiac enzymes (troponin T and CK) were meticulously monitored prior to infusion, at 4-hours post-infusion, and 12-hours post-infusion. The results indicated that these enzymes remained within the normal range. Following a multidisciplinary cardio-oncology discussion, it was deemed unnecessary to measure natriuretic peptides, based on the clinical and echocardiographic findings. Differential considerations for the observed CK and troponin elevations included chemotherapy-induced cardiotoxicity (considered the first hypothesis due to troponin elevation and ECG alterations); myocarditis, excluded due to the absence of fever and normal inflammatory markers; skeletal muscle injury, excluded due to the absence of myalgia; and systemic inflammatory stress, also excluded. These possibilities were evaluated using clinical assessment, serial biomarkers, imaging, and laboratory data.

Instead of block 3, blinatumomab was administered at a dosage of 15 µg/m^2^ per day for a period of 28 days, encompassing two cycles. Following the conclusion of the treatment regimen, molecular MRD monitoring showed a CR, as indicated by the presence of marker 1 TRG Vg3 Jg1.3/2.3 negative with QR = 1x10–^5^ and IGK VK2 JK3 negative with QR = 1x10^-4^. The patient then underwent an allogeneic hematopoietic stem cell transplant (HSCT), which was performed uneventfully. The patient is currently in continuous CR 12 months after HSCT, and no clinical, ECG, echocardiographic, or metabolic cardiologic abnormalities have been observed. The complete timeline and the applied multidisciplinary clinical interventions are displayed in **Table 1**.

## 3 Discussion

Cardiotoxicity is a well-documented complication in oncology patients, particularly in the pediatric population (4–7). The prolonged life expectancy of young survivors underscores the necessity for meticulous monitoring to discern early or late cardiotoxicity patterns. The early detection and management of cardiotoxicity represent a crucial challenge in the prevention of long-term cardiovascular complications. This assertion is supported by a Statement of the American Heart Association, which draws upon both metabolic and clinical perspectives (11, 12). The most significant well-known risk of cardiac complications is associated with anthracycline treatment (particularly, but not exclusively, represented by doxorubicin and daunorubicin), and high-dose cyclophosphamide (4–7). In 2022, the European Society of Cardiology (ESC) published new guidelines that introduced the concept of cancer therapy-related cardiovascular toxicity, emphasizing the importance of identifying useful biomarkers to facilitate early detection of cardiotoxicity (13, 14). Among them, troponins (cardiac troponin T and troponin I) and natriuretic peptides (atrial natriuretic peptide, brain natriuretic peptide, and N-terminal pro-brain natriuretic peptide) have been the focus of study and employed for the purpose of evaluating cancer-therapy-induced cardiotoxicity (6, 7, 13–15). Cardiac troponins are utilized for the identification of structural injury to cardiomyocytes, while natriuretic peptides exhibit high prognostic accuracy for death or hospitalization due to heart failure (15).

CK is a biomarker for both skeletal and cardiac muscle damage, though its clinical interpretation is limited by low specificity. Elevations are linked to physiological factors (age, gender, race, and physical exercise) and various pathological conditions, including neuromuscular disorders, infections, and drug-induced toxicities (9, 16–18). An elevated level of CK could be indicative of toxicity, with the potential to influence the efficacy of the treatment protocol. This, in turn, could result in discontinuations or reductions in dosage.

In this work, we described the case of a child diagnosed with ALL who exhibited two episodes of severe CK elevation during the induction treatment phase. These elevations, although not specific, were potentially associated with cardiac muscle damage, secondary to chemotherapy-induced cardiotoxicity. This hypothesis is supported by the concomitant increase in troponin T values and ECG abnormalities, which resulted in a modification of the treatment schedule. It has been demonstrated that natriuretic peptides and troponins are the most reliable biomarkers for an early diagnosis of chemotherapy-induced cardiotoxicity (6, 7, 13–15). However, it should be noted that limitations are present: troponin levels may not always directly correlate with the degree of damage, and their elevation can occur in a variety of cardiac and non-cardiac conditions, which may limit their specificity in reliably indicating a cardiotoxicity episode. Natriuretic peptides have been observed to increase in conditions such as chronic kidney disease, pulmonary disease, or other systemic stressors that do not involve direct myocardial injury. Furthermore, the levels of these markers may not always be sufficiently sensitive in the early stages of cardiotoxicity, potentially leading to false-negative results in cases of minimal or subclinical damage. Troponins and natriuretic peptides are of significant importance in the diagnosis of cardiotoxicity; however, they should be used in conjunction with other diagnostic tools to achieve a more complete and comprehensive assessment (19–21).

Despite the relatively low specificity and sensitivity of CK, daily monitoring of this enzyme could prove beneficial as a preliminary step for the detection of potential cardiac muscle damage in patients receiving chemotherapeutic treatments, as recently reported (9, 12, 22, 23). To address the need for clinical standardization, we propose an actionable pathway wherein a CK elevation exceeding the upper limit of the reference range, or a significant percentage increase from the patient’s baseline, serves as a diagnostic ‘red flag’. We acknowledge that the current study describes a single case, which inherently limits the generalizability of these findings and the formal validation of specific thresholds. However, prospective studies are underway to initiate a routine evaluation of this parameter in a larger cohort. The incremental value of CK lies in its role as a sentinel for acute cellular stress. An elevation in CK may act as a preliminary signal to trigger further biochemical analyses (CK isoenzymes, cardiac troponins, and natriuretic peptides) and instrumental investigations (ECG, echocardiogram) to confirm or rule out myocardial damage. This is particularly relevant within the 24–72 hour window following chemotherapy administration, where CK monitoring can prompt focused second-level testing. This approach aligns with evidence suggesting that multi-marker strategies significantly improve the negative predictive value for chemotherapy-induced cardiotoxicity (24, 25). Our future research will employ a systematic approach to enzyme elevations, aiming to define precise action pathways and further articulate the clinical utility of CK as a first-line biomarker. In the era of targeted anticancer therapy, mitigating cardiac damage and ensuring patient safety—particularly within the pediatric population—remains a clinical priority.

## Data Availability

The raw data supporting the conclusions of this article will be made available by the authors, without undue reservation.

## References

[1] Lustosa de Sousa DW de Almeida Ferreira FV Cavalcante Felix FH de Oliveira Lopes MV . Acute lymphoblastic leukemia in children and adolescents: prognostic factors and analysis of survival. Rev Bras Hematol Hemoter. (2015) 37:223–9. doi: 10.1016/j.bjhh.2015.03.009. PMID: 26190424 PMC4519710

[2] Pui CH Yang JJ Hunger SP Pieters R Schrappe M Biondi A . Childhood acute lymphoblastic leukemia: progress through collaboration. J Clin Oncol. (2015) 33:2938–48. doi: 10.1200/jco.2014.59.1636. PMID: 26304874 PMC4567699

[3] Inaba H Mullighan CG . Pediatric acute lymphoblastic leukemia. Haematologica. (2020) 105:2524–39. doi: 10.3324/haematol.2020.247031. PMID: 33054110 PMC7604619

[4] Lipshultz SE Miller TL Scully RE Lipsitz SR Rifai N Silverman LB . Changes in cardiac biomarkers during doxorubicin treatment of pediatric patients with high-risk acute lymphoblastic leukemia: associations with long-term echocardiographic outcomes. J Clin Oncol. (2012) 30:1042–9. doi: 10.1200/jco.2010.30.3404. PMID: 22370326 PMC3341148

[5] Mladosievicova B Urbanova D Radvanska E Slavkovsky P Simkova I . Role of NT-proBNP in detection of myocardial damage in childhood leukemia survivors treated with and without anthracyclines. J Exp Clin Cancer Res. (2012) 31:86. doi: 10.1186/1756-9966-31-86. PMID: 23057994 PMC3503876

[6] Antoniadi K Thomaidis N Nihoyannopoulos P Toutouzas K Gikas E Kelaidi C . Prognostic factors for cardiotoxicity among children with cancer: definition, causes, and diagnosis with omics technologies. Diagnostics (Basel). (2023) 13. doi: 10.3390/diagnostics13111864. PMID: 37296716 PMC10252297

[7] Kouwenberg TW van Dalen EC Feijen EAM Netea SA Bolier M Slieker MG . Acute and early-onset cardiotoxicity in children and adolescents with cancer: a systematic review. BMC Cancer. (2023) 23:866. doi: 10.1186/s12885-023-11353-9. PMID: 37710224 PMC10500898

[8] Moghadam-Kia S Oddis CV Aggarwal R . Approach to asymptomatic creatine kinase elevation. Cleve Clin J Med. (2016) 83:37–42. doi: 10.3949/ccjm.83a.14120. PMID: 26760521 PMC4871266

[9] Zhang H To KKW . Serum creatine kinase elevation following tyrosine kinase inhibitor treatment in cancer patients: symptoms, mechanism, and clinical management. Clin Transl Sci. (2024) 17:e70053. doi: 10.1111/cts.70053. PMID: 39473122 PMC11522029

[10] Qiu KY Xu HG Luo XQ Mai HR Liao N Yang LH . Prognostic value and outcome for ETV6/RUNX1-positive pediatric acute lymphoblastic leukemia: a report from the South China Children's Leukemia Group. Front Oncol. (2021) 11:797194. doi: 10.3389/fonc.2021.797194. PMID: 34988026 PMC8722219

[11] Lipshultz SE Adams MJ Colan SD Constine LS Herman EH Hsu DT . Long-term cardiovascular toxicity in children, adolescents, and young adults who receive cancer therapy: pathophysiology, course, monitoring, management, prevention, and research directions: a scientific statement from the American Heart Association. Circulation. (2013) 128:1927–95. doi: 10.1161/cir.0b013e3182a88099. PMID: 24081971

[12] Ryan TD Bates JE Kinahan KE Leger KJ Mulrooney DA Narayan HK . Cardiovascular toxicity in patients treated for childhood cancer: a scientific statement from the American Heart Association. Circulation. (2025) 151:e926–43. doi: 10.1161/cir.0000000000001308. PMID: 40104841 PMC12265934

[13] Zamorano JL Lancellotti P Munoz DR Aboyans V Asteggiano R Galderisi M . 2016 ESC Position Paper on cancer treatments and cardiovascular toxicity developed under the auspices of the ESC Committee for Practice Guidelines. Kardiol Pol. (2016) 74:1193–233. doi: 10.5603/kp.2016.0156. PMID: 27910076

[14] Lyon AR Lopez-Fernandez T Couch LS Asteggiano R Aznar MC Bergler-Klein J . 2022 ESC Guidelines on cardio-oncology developed in collaboration with the European Hematology Association (EHA), the European Society for Therapeutic Radiology and Oncology (ESTRO) and the International Cardio-Oncology Society (IC-OS). Eur Heart J Cardiovasc Imaging. (2022) 23:e333–465. doi: 10.1093/ehjci/jeac106. PMID: 36017575

[15] Zhang Z Zheng Z Nie H Dong H Lei C . Association between the preoperative N-terminal pro-B-type natriuretic peptide and acute kidney injury in gastrointestinal surgery patients managed with enhanced recovery strategy: a retrospective cohort study. Perioper Med (Lond). (2025) 14:45. doi: 10.1186/s13741-025-00528-6. PMID: 40264198 PMC12016297

[16] Parafianowicz P Krishan R Beutler BD Islam RX Singh T . Myositis - a common but underreported adverse effect of osimertinib: case series and review of the literature. Cancer Treat Res Commun. (2020) 25:100254. doi: 10.1016/j.ctarc.2020.100254. PMID: 33276288

[17] Sugimoto H Matsumoto S Tsuji Y Sugimoto K . Elevated serum creatine kinase levels due to osimertinib: a case report and review of the literature. J Oncol Pharm Pract. (2022) 28:489–94. doi: 10.1177/10781552211042271. PMID: 34605320

[18] Sakamoto T Saito Y Takekuma Y Kikuchi E Sugawara M . Gefitinib-induced myositis: a novel case report. Yakugaku Zasshi. (2023) 143:617–20. doi: 10.1248/yakushi.23-00007. PMID: 37394456

[19] Bracun V de Boer RA . Troponins and natriuretic peptides to detect cardiotoxicity: useful biomarkers or paradise lost? Eur J Heart Fail. (2020) 22:362–5. doi: 10.1002/ejhf.1676. PMID: 31944511

[20] Pudil R Mueller C Celutkiene J Henriksen PA Lenihan D Dent S . Role of serum biomarkers in cancer patients receiving cardiotoxic cancer therapies: a position statement from the Cardio-Oncology Study Group of the Heart Failure Association and the Cardio-Oncology Council of the European Society of Cardiology. Eur J Heart Fail. (2020) 22:1966–83. doi: 10.1002/ejhf.2017. PMID: 33006257

[21] Attanasio U Di Sarro E Tricarico L Di Lisi D Armentaro G Miceli S . Cardiovascular biomarkers in cardio-oncology: antineoplastic drug cardiotoxicity and beyond. Biomolecules. (2024) 14. doi: 10.3390/biom14020199. PMID: 38397436 PMC10887095

[22] Bankar A Lipton JH . Association of creatine kinase elevation with clinical outcomes in chronic myeloid leukemia: a retrospective cohort study. Leuk Lymphoma. (2022) 63:179–88. doi: 10.1080/10428194.2021.1971219. PMID: 34493150

[23] Lazar DR Farcas AD Blag C Neaga A Zdrenghea MT Cainap C . Cardiotoxicity: a major setback in childhood leukemia treatment. Dis Markers. (2021) 2021:8828410. doi: 10.1155/2021/8828410, PMID: 33505537 PMC7810535

[24] Zardavas D Suter TM de Azambuja E . Biomarkers for the early detection of chemotherapy-induced cardiotoxicity: the search for the "Holy Grail. Cancer Treat Rev. (2015) 41:765–74.

[25] McGowan JV Chung R Maulik A Piotrowska I Malcolm Walker J Yellon DM . Anthracycline-induced cardiotoxicity: causes, mechanisms, and prevention. Cardiovasc Drugs Ther. (2017) 31:63–75. doi: 10.1007/s10557-016-6711-0, PMID: 28185035 PMC5346598

